# The effect of dipyridamole on the pharmacokinetics of metformin: a randomized crossover study in healthy volunteers

**DOI:** 10.1007/s00228-016-2039-8

**Published:** 2016-03-15

**Authors:** S. El Messaoudi, F. G. Russel, A. Colbers, C. C. J. G. Bandell, P. H. H. van den Broek, D. M. Burger, G. A. Rongen, N. P. Riksen

**Affiliations:** Department of Pharmacology-Toxicology, Radboud University Medical Center, Nijmegen, The Netherlands; Department of Pharmacy, Radboud University Medical Center, Nijmegen, The Netherlands; Department of Internal Medicine 463, Radboud University Medical Center, PO Box 9101, 6500 HB Nijmegen, The Netherlands

**Keywords:** Metformin, Dipyridamole, ENT4, PMAT, Drug interaction, Pharmacokinetics

## Abstract

**Purpose:**

Concomitant treatment with the glucose-lowering drug metformin and the platelet aggregation inhibitor dipyridamole often occurs in patients with type 2 diabetes mellitus who have suffered a cerebrovascular event. The gastrointestinal uptake of metformin is mediated by the human equilibrative nucleoside transporter 4 (ENT4), which is inhibited by dipyridamole in preclinical studies. We hypothesized that dipyridamole lowers the plasma exposure to metformin.

**Methods:**

Eighteen healthy volunteers (mean age 23 years; 9 male) were randomized in an open-label crossover study. Subjects were allocated to treatment with metformin 500 mg twice daily in combination with dipyridamole slow-release 200 mg twice daily or to metformin alone for 4 days. After a washout period of 10 days, the volunteers were crossed over to the alternative treatment arm. Blood samples were collected during a 10-h period after intake of the last metformin dose. The primary endpoint was the area under the plasma concentration-time curve (AUC_0–12h_) and the maximum plasma metformin concentration (*C*_max_).

**Results:**

In healthy subjects, dipyridamole did not significantly affect C_max_ nor AUC_0–12h_ of metformin under steady-state conditions.

**Conclusions:**

Previous in vitro studies report that dipyridamole inhibits the ENT4 transporter that mediates gastrointestinal uptake of metformin. In contrast, co-administration of dipyridamole at therapeutic dosages to healthy volunteers does not have a clinically relevant effect on metformin plasma steady-state exposure. This observation is reassuring for patients who are treated with this combination of drugs.

**Electronic supplementary material:**

The online version of this article (doi:10.1007/s00228-016-2039-8) contains supplementary material, which is available to authorized users.

## Introduction

Patients with type 2 diabetes mellitus are at increased risk for cardiovascular disease and associated cardiovascular events [1]. The incidence of ischemic stroke is significantly increased in these patients [2]. Metformin, a biguanide glucose-lowering agent, is the first line oral treatment option in patients with type 2 diabetes mellitus [3]. The primary mechanism of action is inhibition of hepatic gluconeogenesis [4]. In addition, there is preclinical and clinical evidence that metformin has direct cardiovascular protective properties independent of its glucose-lowering action [5].

Dipyridamole is registered for the secondary prevention of cerebral ischemia in combination with acetylsalicylic acid, since studies have shown that dipyridamole added to acetylsalicylic acid reduces the incidence of myocardial infarction, stroke, or cardiovascular death [6]. Therefore, concomitant treatment with metformin and dipyridamole often occurs in patients with type 2 diabetes who have suffered a cerebrovascular event, and potential pharmacokinetic interactions between these drugs are highly relevant.

Metformin is a highly hydrophilic compound with a net positive charge at physiologic pH and its absorption, distribution, and elimination are dependent on active transport by several carrier-mediated transporters [7]. The uptake of metformin from the gastrointestinal tract is mediated, at least in part, by the human equilibrative nucleoside transporter 4 (ENT4; gene *SLC29A4*), also known as the plasma membrane monoamine transporter (PMAT) [7, 8]. ENT4 is localized on the apical membrane of enterocyte villus tips and exhibits great sensitivity to pH, with an optimal activity at a pH of approximately 6.0 [8, 9]. Renal excretion of metformin is mediated by the human organic cation transporter hOCT2, which is expressed on the basolateral membrane of proximal tubular cells, and by multidrug and toxin extrusion transporters (MATE1 and to a lesser extent MATE2K), which are present in the apical membrane [7]. Uptake into hepatocytes occurs via hOCT1 and hOCT3, and excretion into bile is mediated by MATE1 [7, 10]. This is corroborated by the findings of a profound reduction in the apparent volume of distribution of metformin in OCT1- and OCT3-knockout mice [11, 12].

The transport characteristics of ENT4 have been studied in various in vitro models [9, 13]. Substrate transport by ENT4 (tested for adenosine and 1-methyl-4-phenylpyridinium) is significantly inhibited by dipyridamole with an IC50 value of approximately 5 μM, which is in the range of the plasma concentration during normal therapeutic use of dipyridamole [14]. Therefore, by inhibition of the apical ENT4, concomitant treatment with dipyridamole might result in a lower bioavailability and plasma concentration of metformin.

The aim of this study was to determine whether concomitant use of dipyridamole reduces the gastrointestinal absorption of metformin in healthy subjects.

## Methods

### Participants

Healthy male and female participants between the ages of 18 and 50 years were eligible for enrollment. The included participants had to be in good health, free from cardiovascular disease, diabetes mellitus and hypertension (systolic blood pressure ≥140 mmHg and/or diastolic ≥90 mmHg), and non-smoking. We excluded individuals with renal dysfunction (GFR MDRD <60 ml/min/1.73 m^2^), ECG abnormalities, and those who were taking concomitant medication. Oral contraception use by female participants was permitted.

### Ethics statement

The study was approved by the local ethics committee and was performed in the Radboud University Medical Center in compliance with the recommendations of the Declaration of Helsinki. We obtained written informed consent from all volunteers before participation. Preparation, conduct, and analysis of this trial complied with Good Clinical Practice guidelines. This trial was prospectively registered at www.clinicaltrials.gov (NCT01613755).

### Experimental design

In a prospective randomized open-label two-period crossover study, subjects were allocated to treatment with either metformin hydrochloride 500 mg (Mylan, Bunschoten, The Netherlands) twice daily in combination with dipyridamole slow release 200 mg (Boehringer Ingelheim, Alkmaar, The Netherlands) twice daily, both for 4 days to ensure a steady-state plasma concentration, or to metformin 500 mg alone twice daily for 4 days. After a washout period of 10 days, the volunteers were crossed over to the alternative treatment arm. An independent researcher performed simple random allocation by the use of sealed envelopes. We studied the interaction between metformin and dipyridamole at steady-state for both drugs, because this more accurately reflects common clinical practice. To demonstrate that a steady-state plasma concentration was reached, blood was drawn in the morning of the third treatment day and fourth day, immediately before intake of the morning dose of dipyridamole and 1 h later, which was just before the morning dose of metformin, to determine the trough plasma concentration of dipyridamole and metformin, respectively. On the fourth treatment day, the subjects attended our research center for the pharmacokinetic studies. All subjects had to abstain from alcohol consumption for at least 24 h before the measurements, since alcohol can inhibit ENT transport [15]. The morning doses of both metformin and dipyridamole on the days of serial blood sampling were given under supervision at our research center. To ensure optimal dipyridamole exposure to the gastrointestinal ENT transporters, the dipyridamole dose was administered 1 h before the metformin dose. After intake of the dipyridamole dose, the volunteers were allowed to have breakfast. Lunch was served no earlier than 4 h after metformin dosing and participants received a snack no earlier than 2 h after lunch.

### Pharmacokinetic sampling and safety assessments

Blood samples for the assessment of pharmacokinetic parameters of metformin were collected during a 10-h period at *t* = 0 (pre-dose), 1, 1.5, 2, 2.5, 3, 3.5, 4, 5, 6, 8, and 10 h after the intake of the morning dose of metformin. These time points were chosen because the *T*_max_ of metformin is approximately 2.5 h. Blood samples were collected in heparinized tubes and centrifuged for 10 min at 3000 rpm (1700*g*) at 20 °C. Plasma was transferred to polypropylene tubes and stored at −80 °C until further analysis. Subjects were asked about the occurrence of adverse events at each visit day.

### Compliance

Participants were asked to return the package of their study medication on both study days. Compliance to medication was monitored by counting the remaining tablets on each study day.

### Analysis of metformin and dipyridamole concentrations in plasma

See supplement.

### Pharmacokinetic analysis

Pharmacokinetic parameters for metformin were calculated by noncompartmental methods using the WinNonlin software package (version 6.3; Pharsight, Mountain View, CA) and the linear log-trapezoidal rule. Based on the individual plasma concentration-time data, the following pharmacokinetic parameters of metformin were determined: AUC_0–10h_, the extrapolated AUC from 0 to 12 h after intake (AUC_0–12h_), *C*_max_, *T*_max_, V/F, CL/F and the apparent elimination half-life (*T*_1/2_). *C*_12h_ was extrapolated by log-linear extrapolation using lambda *z* from the last measured concentration (*C*_10h_). Pharmacokinetic parameters are reported as geometric means with CV%. Geometric mean ratios (GMRs) of the pharmacokinetic parameters of the test treatment [combination metformin + dipyridamole] versus the reference treatment [metformin alone] and 90 % CI were calculated after log transformation of within-subject ratios using a mixed effects bioequivalence module in WinNonlin/Phoenix.

### Sample size and statistical analysis

For the identification of a clinically relevant drug interaction, the bioequivalence approach was used, as described previously [16]. The main pharmacokinetic parameter to be evaluated in this respect is the exposure to metformin, expressed as AUC_0–12h_. This pharmacokinetic parameter and the *C*_max_ are considered to be the primary measures in this study. Sample size calculation was performed using the method for two-period designs of Diletti et al. [17]. The required sample size was calculated (power of 80 %) assuming no difference in the AUC of metformin with or without dipyridamole and an estimated intra-subject coefficient of variation of the AUC values for metformin of 19 %, as reported in other pharmacokinetic studies with metformin in healthy volunteers [7, 18]. The required number of participants was 16. Taking dropouts into account, a total of 18 subjects were included.

## Results

### Baseline characteristics

We included 18 subjects. One participant withdrew during the study due to difficulties with venous sampling. This participant was only included in the safety analysis. Therefore, 17 subjects finished the trial protocol (Suppl. Fig. 1) and were included in the analysis of pharmacokinetic parameters. Baseline characteristics are presented in Table 1.

**Table 1:** Baseline characteristics

Age, years	23 (range 20–25)
Gender	
Male	9 (53 %)
Female	8 (47 %)
Body mass index, kg/m^2^	22.1 ± 3.0
Blood pressure, mmHg	
Systolic	131 ± 9
Diastolic	80 ± 6
Heart rate, beats/min	68 ± 8
Smoking	0 (0 %)
Alcohol consumption	
≤2 units per day	15 (88 %)
>2 units per day	2 (12 %)
Laboratory values at screening	
Glucose, mmol/L	5.1 ± 1.0
Creatinin, μmol/L	73 ± 13
GFR (MDRD), ml/min/1.73 m^2^	81 ± 14

Data are mean ± SD (except for age, mean (range)) or number (%) unless stated otherwise

*GRF* glomerular filtration rate, *MDRD* modification of diet in renal disease

### Compliance

The compliance with both metformin and dipyridamole treatment of all subjects was excellent, as indicated by their statements about the intake of the drug doses and the number of tablets counted on each visit day. All subjects took their study medication according to protocol, without missing a dose.

### Evaluation of steady state

Mean (SD) trough plasma concentrations of both metformin and dipyridamole collected on the third treatment day and the morning of the fourth treatment day (day of serial blood collection) showed that subjects were adherent and that steady state conditions for both metformin and dipyridamole were reached (Supplementary Table 1).

### Metformin pharmacokinetics

**Fig. 1:**
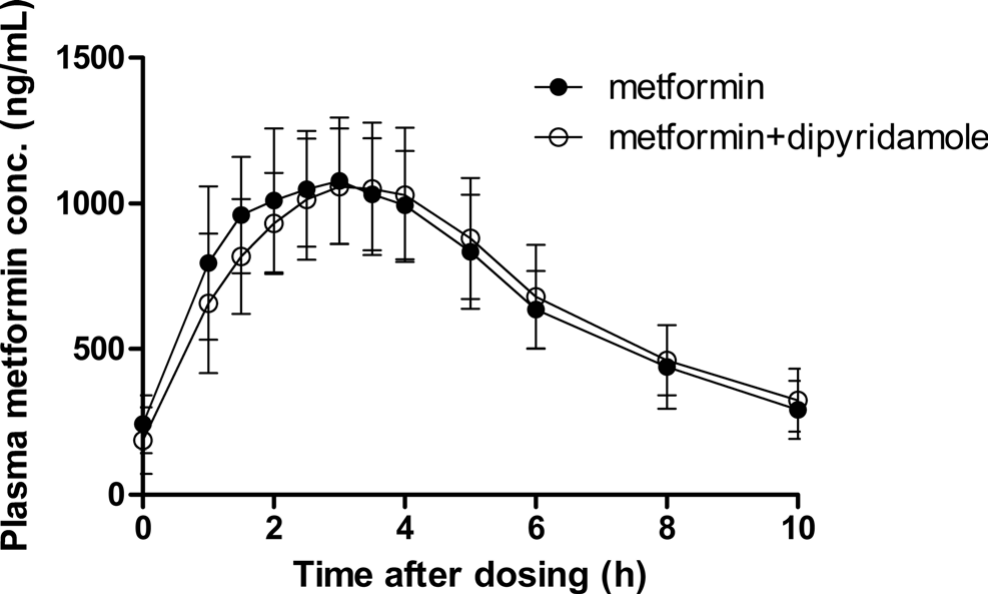
Plasma metformin concentrations (geometric mean ± CV%)

The pharmacokinetic parameters and the plasma concentration-time curves of metformin in the presence and absence of dipyridamole are shown in Table 2 and Fig. 1. All 90 % confidence intervals fell within the 80–125 % bioequivalence criterium. This means that dipyridamole did not affect *C*_max_ and AUC_0–12h_ of metformin.

**Table 2:** Comparison of steady state pharmacokinetic parameters of metformin with or without co-administration of dipyridamole to healthy volunteers

Pharmacokinetic parameter	Metformin (GM (CV%))	Metformin + dipyridamole (GM (CV%))	GMR (90 % CI)
AUC_0-12h_ (ng×h/mL)	7600 (19)	7600 (19)	100 (93–108)
AUC_0-10h_ (ng×h/mL)	7100 (18)	7000 (19)	99 (93–107)
CL/F (L/h)	66 (19)	66 (19)	100 (93–107)
V/F (L) Winnonlin	340 (26)	370 (40)	108 (96–120)
T_1/2, desc_ (h) Winnonlin	3.6 (25)	3.9 (36)	108 (99–118)
T_max_ (h)	3 (1–4)	3.5 (2.5–6)	
C_max_ (ng/mL)	1100 (19)	1100 (19)	101 (95–107)

Results of noncompartmental analysis; for *T*_max,_ median and range are reported

*GM* geometric mean

### Adverse events and safety assessments

The study medication was generally well tolerated, and no serious adverse events occurred. In the metformin-dipyridamole treatment arm, 13 subjects (77 %) reported headache and 8 subjects (47 %) reported mild, self-limiting gastrointestinal discomfort, mostly diarrhea. One person developed a phlebitis of his arm after removal of the cannula. In the metformin-only group, 8 subjects (47 %) reported mild and self-limiting gastrointestinal discomfort, mostly diarrhea. There was no headache reported in this group. One subject reported the occurrence of vivid nightmares, considered not related to the study medication. Adverse events occurred mostly within several hours after start of the study medication, were mild (all were classified as grade 1), and self-limiting.

## Discussion

We tested the hypothesis that dipyridamole reduces the bioavailability of metformin after oral administration by inhibition of the luminal ENT4 in the small intestine. In a classical randomized crossover pharmacokinetic interaction study in healthy subjects, there was no effect of co-administered slow-release dipyridamole on *C*_max_ and AUC_0–12h_ of metformin. As such, we can conclude that concomitant treatment with slow-release dipyridamole does not relevantly affect the plasma exposure to metformin. This finding is of great importance since many patients with type 2 diabetes mellitus who have suffered a stroke or transient ischemic attack are being treated with both of these drugs.

Our hypothesis that dipyridamole limits intestinal absorption of metformin after oral intake was driven by the following observations. First, ENT4 is important in the luminal uptake of metformin [7, 8]. Secondly, dipyridamole significantly inhibits ENT4-mediated substrate transport with relevant IC50 values [14]. Thirdly, genetic variations in the gene encoding ENT4 affect steady-state plasma levels of metformin, emphasizing that this transporter is important for metformin uptake [19]. Based on these findings, concomitant use of metformin and dipyridamole may result in lower systemic exposure to metformin which may impact on serum glucose.

In contrast to our hypothesis, we observed no significant impact of dipyridamole on plasma exposure to metformin in clinical relevant doses, as reflected by AUC_0–12h_ and *C*_max_. There are several potential explanations as to why dipyridamole does not limit intestinal absorption of metformin. First, a reduced ENT4-mediated uptake of metformin might be compensated for by an increased absorption by alternative intestinal transporters that are not affected by dipyridamole. Human small intestine contains detectable messenger RNA (mRNA) expression of OCT1-3, OCTN1-2, and ENT4 [8, 20, 21]. Based on earlier literature, only OCT3 and ENT4 appear to be localized on the apical border of the enterocytes and could therefore play a role in intestinal absorption. More recently, apical expression of the serotonin reuptake transporter (SERT) and OCTN1 in enterocytes was demonstrated, which may also transport metformin [22–24]. An alternative explanation was provided by Proctor et al., who reported significant paracellular uptake of metformin [25]. These experiments, however, were performed in vitro in Caco-2 cells, which differ importantly from the physiological in vivo situation in human small intestine. Finally, although the IC50 value of the ENT4 for dipyridamole is in the concentration range that is obtained in plasma after oral dipyridamole administration, we are not informed about the luminal concentration of dipyridamole at the site of metformin absorption. A potential mechanism that could increase absorption of metformin could be the modulation of the intraluminal pH by dipyridamole: the extended release preparation of dipyridamole contains tartaric acid. As dipyridamole is a poorly soluble weak base that shows pH-dependent absorption, this formulation should provide a sufficiently acidic local milieu within the basic pH environment of the intestine to promote absorption [26]. Theoretically, this could have been favorable for ENT4-mediated metformin transport, which is greatly stimulated by acidic pH [8].

Study medication was well tolerated and subjects experienced only mild side effects. The occurrence of headache was associated with use of dipyridamole, while gastrointestinal discomfort was related to metformin [27, 28]. This mainly involved mild and self-limiting diarrhea in the first days of metformin treatment. We think it is unlikely that a potentially shorter gastrointestinal passage time relevantly affected the results of our study, since diarrhea occurred to a similar extent in both treatment arms, and in most patients, it was restricted to the first few days of metformin intake.

There are a few potential limitations of our study. First, genetic variation in the genes encoding for the various metformin transporters, including ENT4 and OCT1-2, have been linked previously to the altered pharmacokinetic and pharmacodynamic response to metformin, but we did not perform any genotyping of the relevant transporters. Secondly, we did not collect urine from the subjects. This would have enabled us to more directly estimate the effect of dipyridamole on metformin elimination.

In conclusion, co-administration of dipyridamole at therapeutic dosages to healthy volunteers does not have a clinically relevant effect on metformin plasma steady-state exposure. This observation is reassuring for patients who are treated with this combination of drugs.

## Electronic supplementary material

Below is the link to the electronic supplementary material.

ESM 1 (DOCX 16 kb)

ESM 2 (DOCX 18 kb)
