# Supplementary material for: The effect of dipyridamole on the pharmacokinetics of metformin: a randomized crossover study in healthy volunteers
Source: Eur J Clin Pharmacol. 2016 Mar 15;72:725–30. doi: 10.1007/s00228-016-2039-8 (PMC4869751; doi:10.1007/s00228-016-2039-8)
Supplement: Supplementary file 1 — (DOCX 16 kb) [file 228_2016_2039_MOESM1_ESM.docx]

**The effect of dipyridamole on the pharmacokinetics of metformin: a randomized cross-over study in healthy volunteers**

S. El Messaoudi, F.G. Russel, A. Colbers, C.C.J.G. Bandell, P.H.H. van den Broek, D.M. Burger, G.A. Rongen, N.P. Riksen

**SUPPLEMENTARY METHODS AND RESULTS**

*Analysis of metformin and dipyridamole concentrations in plasma*

After removing phospholipids and proteins with HybridSPE-phospholipids columns (Supelco), the plasma metformin concentration was determined with LC-MS/MS, using an Accela U-HPLC (Thermo Fischer Scientific) coupled to a TSQ Vantage (Thermo Fisher Scientific) triple quadropole mass spectrometer. The compounds were separated on a Zorbax HILIC Plus (100 x 2.1 mm, 3.5 µm particle size; Agilent Technologies). As internal standard we used metformin-d6 (Toronto Research Chemicals Inc.) The elution gradient was as follows: 0 min, 100% B; 5 min, 50% B; and 6 min, 100% B. Solvent A consisted of 2 mM NH4 formate+0.1% formic acid in H2O and Solvent B consisted of 2 mM NH4 formate+0.1% formic acid in 90% acetonitril. The column temperature was set at 40°C, and the flow rate was 200 µl/min. The effluent from the U-HPLC was passed directly into the electrospray ion source. Positive electrospray ionization was achieved using a nitrogen sheath gas with ionization voltage set at 3500 Volt. The capillary temperature was set at 290°C. Detection of metformin and the internal standard was based on isolation of the protonated molecular ion, [M+H]+ and subsequent MS/MS fragmentations and a selected reaction monitoring (SRM) were carried out. The following SRM transitions were used: for metformin *m/z* 130.1 (parent ion) to *m/z* 60.1 and 71.1 (both product ions) and for metformin-d6 *m/z* 136.1 (parent ion) to m/z 77.1 (product ion). The range of the calibration curve for metformin was 0-1200 ng/ml, with an R^2^=1.000.

Plasma dipyridamole concentrations were determined in deproteinized plasma by use of HPLC with fluorescence detection set at 286 nm/470 nm as previously described by Wolfram and Bjornsson [1]. The range of the calibration curve for dipyridamole was 0-4 µg/ml, R^2^=0.997. All measurements were performed in duplicate, and the intra-assay coefficient of variation was less than 10%.

**Supplementary Table 1:** Trough plasma concentrations of metformin and dipyridamole.

|  | Metformin (MET only) (ng/mL) | Metformin (MET-DIPY) (ng/mL) | Dipyridamole  (µg/mL) |
| --- | --- | --- | --- |
|  |  |  |  |
| Day 3 | 390 ± 350 | 260 ± 130 | 1.4 ± 0.5 |
| Day 4 | 250 ± 140 | 230 ± 110 | 1.3 ± 0.4 |
|  |  |  |  |

Data are presented as mean ± SD. MET: metformin, DIPY: dipyridamole.

*References*

1 Wolfram KM, Bjornsson TD (1980) High-performance liquid chromatographic analysis of dipyridamole in plasma and whole blood. *J Chromatogr* 183 (1): 57-64
