# Supplementary material for: The effect of dipyridamole on the pharmacokinetics of metformin: a randomized crossover study in healthy volunteers
Source: Eur J Clin Pharmacol. 2016 Mar 15;72:725–30. doi: 10.1007/s00228-016-2039-8 (PMC4869751; doi:10.1007/s00228-016-2039-8)
Supplement: Supplementary file 2 — (DOCX 18 kb) [file 228_2016_2039_MOESM2_ESM.docx]

**Suppl. Fig. 1**

## Follow-Up

## Allocation

## Analysis

Allocated to metformin only (n= 9) in first period

♦ Received allocated intervention (n= 9)

Allocated to metformin and dipyridamole

(n= 9) in first period

♦ Received allocated intervention (n= 9)

♦ Did not complete experiment (n= 1) due to difficulties with venous sampling

Metformin only in 2^nd^ period (n= 8)

♦ Received allocated intervention (n= 8)

Metformin and dipyridamole (n= 9) in 2^nd^ period

♦ Received allocated intervention (n= 9)

Lost to follow-up (n= 0)

Discontinued intervention (n= 0) )

Lost to follow-up (n= 0)

Discontinued intervention (n= 0)

Analysed (n= 8)
♦ Excluded from analysis (n= 0)

Analysed (n= 9)
♦ Excluded from analysis (n= 0)

Excluded (n= 0)

## Enrollment

Randomized (n= 18)

Assessed for eligibility (n= 18)
